# Regulating Cytoplasmic Calcium Homeostasis Can Reduce Aluminum Toxicity in Yeast

**DOI:** 10.1371/journal.pone.0021148

**Published:** 2011-06-15

**Authors:** Xuan Li, Jia Qian, Chaoqun Wang, Ke Zheng, Lan Ye, Yu Fu, Ning Han, Hongwu Bian, Jianwei Pan, Junhui Wang, Muyuan Zhu

**Affiliations:** 1 State Key Laboratory of Plant Physiology and Biochemistry, Key Laboratory for Cell and Gene Engineering of Zhejiang Province, College of Life Sciences, Zhejiang University, Hangzhou, China; 2 College of Chemistry and Life Sciences, Zhejiang Normal University, Jinhua, China; Roswell Park Cancer Institute, United States of America

## Abstract

Our previous study suggested that increased cytoplasmic calcium (Ca) signals may mediate aluminum (Al) toxicity in yeast (*Saccharomyces cerevisiae*). In this report, we found that a yeast mutant, *pmc1*, lacking the vacuolar calcium ion (Ca^2+^) pump Ca^2+^-ATPase (Pmc1p), was more sensitive to Al treatment than the wild-type strain. Overexpression of either PMC1 or an anti-apoptotic factor, such as Bcl-2, Ced-9 or PpBI-1, decreased cytoplasmic Ca^2+^ levels and rescued yeast from Al sensitivity in both the wild-type and *pmc1* mutant. Moreover, pretreatment with the Ca^2+^ chelator BAPTA-AM sustained cytoplasmic Ca^2+^ at low levels in the presence of Al, effectively making the cells more tolerant to Al exposure. Quantitative RT-PCR revealed that the expression of calmodulin (CaM) and phospholipase C (PLC), which are in the Ca^2+^ signaling pathway, was down-regulated under Al stress. This effect was largely counteracted when cells overexpressed anti-apoptotic Ced-9 or were pretreated with BAPTA-AM. Taken together, our results suggest that the negative regulation of Al-induced cytoplasmic Ca signaling is a novel mechanism underlying internal resistance to Al toxicity.

## Introduction

Aluminum (Al) toxicity has been implicated as a major cause of severe loss of crops grown in acidic soils. In recent years, significant progress has been made in understanding the molecular mechanisms of Al toxicity and tolerance in the area of stress phytophysiology. Al targets multiple cellular sites, resulting in disruption of the structure and function of the cell wall, plasma membrane, cytoskeleton, signal transduction pathways, and calcium (Ca) uptake capability [1], [2].

Al exclusion and intracellular resistance are two important mechanisms that promote Al tolerance in plants. External mechanisms defined to date include the release of organic acid anions and phenolic compounds, increased pH in the rhizosphere, modifications in cell wall components, and redistribution and internalization of Al. Organic acid anions, such as citrate, oxalate, and malate, are secreted by roots in response to Al [2], [3]. Some of the genes involved in Al-induced organic acid secretion have been identified as *ALMT1*, *HvAACT1* and *SbMATE* [4], [5], [6]. The expression of the Al-inducible *Arabidopsis* genes *ALS1* and *ALS3* or the rice genes *STAR1*, *STAR2* and *ART1* can confer Al tolerance [7], [8], [9], [10]. Internal resistance can be achieved by both complexation and sequestration. Al is taken up in the ionic form through a transporter and then chelated with organic anions to form a stable, non-phytotoxic complex. Finally, Al becomes sequestrated in the vacuoles in its chelated form [2]. Recently, Nrat1 (Nramp aluminum transporter 1) was identified as a plasma membrane-localized transporter that mediates sequestration of Al into vacuoles in rice [11]. It is important to note that internal resistance to Al can also be achieved through negative regulation of programmed cell death (PCD) [12], [13].

Apoptosis, a typical form of PCD, facilitates the rapid removal of potentially threatening or undesired cells and plays a central role in normal development and homeostasis of metazoan organisms [14], [15], [16]. As in metazoan cells, yeast apoptosis can be detected by typical hallmarks [17]. Bcl-2-family proteins and Bax inhibitor-1 (BI-1) are well known for their ability to respond to stress signals and protect cells against apoptosis [18], [19], [20]. Al is capable of inducing apoptosis in various cell types. Al-induced apoptosis can be inhibited by the up-regulation of Bcl-2 family members or BI-1 [12], [13], [21]. Al increases cytosolic calcium ion (Ca^2+^) levels in yeast, which can be decreased by overexpressing anti-apoptotic members [13]. It remains unknown, however, whether negative regulation of the Ca signals is a significant mechanism involved in internal resistance to Al toxicity.

Abiotic and biotic stresses induce cell death via the disruption of Ca homeostasis in yeast, plant, and animal cells [22], [23], [24]. The Ca^2+^, as a vital intracellular second messenger, governs countless cellular functions. To maintain basal levels of cytoplasmic Ca^2+^ under various and ever-changing conditions, cells have evolved mechanisms to carefully regulate Ca^2+^ entry and removal [25], [26], [27]. Al toxicity strongly affects intracellular Ca homeostasis, which is another mechanism that is hypothesized to cause Al injury [1], [13]. The sensitivity and response of cells to various stresses, including Al, is dependent on the ability of cells to adequately sequester Ca^2+^ into their internal stores [28], [29], [30]. Ca^2+^ is stored in the cell wall and in intracellular organelles, including the endoplasmic reticulum (ER), mitochondria, and vacuoles. In plant and yeast cells, the vacuole serves as the principal Ca^2+^ sequestration site and contains >95% of total cellular Ca^2+^ stores [31], [32], [33]. In yeast, this large Ca^2+^ store is maintained through the action of two specialized transporters: the high-affinity Ca^2+^-ATPase Pmc1p and the low-affinity Ca^2+^/H^+^ exchanger Vcx1p [25], [34]. The deletion of the *PMC1* gene effectively decreases the ability of yeast cells to grow in high Ca^2+^ environments, whereas the deletion of *VCX1* decreases tolerance to Ca^2+^ only slightly; these findings suggest that Pmc1p plays a more significant role in vacuolar Ca^2+^ sequestration.

Pmc1p has approximately 40% identity to mammalian plasma membrane Ca^2+^-ATPases (PMCAs) and functions as a P-type ion pump. Calcineurin activation by Calmodulin (CaM) and elevated cytosolic Ca^2+^ leads to increased expression of PMC1 [35]. CaM is a small acidic protein that contains four EF-hands and is one of the best characterized Ca^2+^ sensors [36]. In yeast, many transcriptional and translational events downstream of the Ca^2+^ signaling pathways are controlled by the Ca^2+^-mediated activation of CaM. CaM is essential for all eukaryotic life and participates in Ca^2+^-dependent stress response pathways through activation of CaM kinases *Cmk1* and *Cmk2* and the phosphatase calcineurin [29]. Phosphoinositide-specific phospholipase C (PLC) is responsible for the production of two second-messenger molecules, containing an activator of protein kinase C diacylglycerol (DAG) and inositol 1,4,5-trisphosphate (IP_3_), which in turn releases Ca from internal stores [37], [38].

Al can induce both PCD and Ca burst in yeast, and anti-apoptotic members can enhance Al tolerance coincident with decreased Ca signals [13]. Therefore, understanding the mechanisms through which cytoplasmic Ca^2+^ regulates Al stress will provide insights into the process of reducing Al toxicity in plants. To this end, we investigated the functional roles of Pmc1p, the Ca^2+^ chelator BAPTA-AM and anti-apoptotic members in modulating cytosolic Ca^2+^ and facilitating Al tolerance.

## Materials and Methods

### Yeast strains, media, and growth conditions

The budding yeast wild-type strain K601/W303-1A (*Mat*a *ade2-1 can1-100 his3-11,15 leu2-3112 trp1-1 ura3-1*) and its isogenic derivative mutants K605 (*pmc1::TRP1*), K661 (*vcx1△*) and K609 (*pmr1::HIS3*) [39] were generously provided by Dr. Kyle W. Cunningham (Johns Hopkins University, Baltimore, MD, USA). Another isogenic derivative mutant K616 (*pmr1::HIS3 pmc1::TRP1 cnb1::LEU2*) [40] was generously provided by Dr. Michael G. Palmgren (University of Copenhagen, Denmark). Yeast cells were grown in YPD (1% yeast extract, 2% bacto-peptone, and 2% glucose), 1/2 SD/Gal-Raf/-Ura or 1/2 SD/Gal-Raf/-His (Clontech, Mountain View, CA, USA) supplemented with 2% agar. AlCl_3_ and CaCl_2_ stock solutions at concentrations of 1 M and 2.5 M, respectively, were filter sterilized and added to liquid medium at room temperature or to plates at <50°C. For the BAPTA-AM test, the cells were incubated with BAPTA-AM (Dojindo Laboratories, Kumamoto, Japan) in liquid medium for 30 min, and then other stresses were added.

### Growth and survival tests

Cells were preincubated in the appropriate liquid medium and allowed to grow twice to the exponential phase. For growth assays, the concentration at OD_600_ was adjusted to 0.05, and then grown with shaking at 200 rpm at 30°C. For spot assays, the OD_600_ of each cell culture was adjusted to 1, and diluted in a 10-fold series (1∶1, 1∶10, 1∶100, 1∶1,000, 1∶10,000); aliquots (5 µL) of each dilution were spotted onto a 1/2 SD/Gal-Raf/-Ura or 1/2 SD/Gal-Raf/-His plate with or without treatment. Plates were incubated at 30°C for 3 days. Cell survival was evaluated by counting colony-forming units (cfu). The cultured cells were harvested at defined intervals, diluted to cell density of 0.0005 at OD_600_, and a 30 µL aliquot of each was plated onto YPD plates [13].

### Constructs and transformation

The yeast-inducible expression vector pYES2 [40] was kindly provided by Prof. Lone Bækgaard (The Royal Veterinary and Agricultural University, Copenhagen, Denmark). To clone the entire coding region of *PMC1*, we designed primers to *PMC1*-P1 (5′-CCC AAG CTT ATG TCT AGA CAA GAC GAA AAT TC-3′) with a *Hind*III site and *PMC1*-P2 (5′-CGC GGA TCC TTA ATA AAA GGC GGT GGA-3′) with a *BamH*I site. The resulting fragment was inserted into the pYES2 vector, in order to express *PMC1* under the control of the *GAL1* promoter and generated the plasmid pYES2-*PMC1*. After confirmation of the fidelity of the constructs by sequencing, the wild-type and *pmc1* mutant yeast strains were, respectively, transformed with plasmids pYES2-*PMC1* and the pYES2 vector by the lithium acetate (LiAc)/polyethylene glycol method [41]. Transformants were selected for uracil prototrophy by plating on 1/2 SD/Glu/-Ura (Clontech) medium. *Bcl-2*, *Ced-9* and *PpBI-1* were inserted into the yeast-inducible expression vector pGilda, which expresses LexA fusion proteins [13]. The three constructs (pGilda-*Bcl-2*, pGilda-*Ced-9*, and pGilda-*PpBI-1*) or the empty vector (pGilda) were transformed into the wild-type yeast and the *pmc1* mutant. Transformants were selected for histidine prototrophy by plating on 1/2 SD/Glu/-His (Clontech) medium.

### RNA isolation, RT-PCR and quantitative RT-PCR

To examine the expression pattern of transformants, the overnight cultures were harvested. The frozen cells were mechanically disrupted using a ball mill [42]. Yeast total RNA was isolated using TRIzol reagent (Invitrogen, Mountain View, CA, USA). To eliminate genomic DNA contamination, an additional DNase treatment was performed with RNase-free DNase (Takara Bio Inc., Shiga, Japan). The extracted RNA was quantified using the BioPhotometer (Eppendorf, Hamburg, Germany). One microgram of total RNA was used for first-strand cDNA synthesis using a PrimeScript RT reagent kit (Takara) and following the manufacturer's instructions.

0.5 µL of cDNA was used as a template for the PCR amplification of target genes, and *ACT1* was used as an internal control (27 cycles). PCR was performed with an initial incubation at 94°C for 5 min, followed by 27 cycles at 94°C for 30 s, 60°C for 30 s, and 72°C for 30 s. The reaction was terminated by a final incubation at 72°C for 10 min. Primers for *LexA* are shown in Table 1.

**Table 1:** Primers used for RT-PCR.

Gene	Primer sequences (5′-3′)^a^	Product size (bps)
*LexA*	F: GCA GGA AGA GGA AGA AGG GTT	182
	R: AGT CAC CAT CCA TAA TGC CGA	
*ACT1*	F: TAC TCT TTC TCC ACC ACT GCT GA	148
	R: CTT GAC CAT CTG GAA GTT CGT AG	
*PMC1*	F: GAG AAT CTG CCC CGA TGA AG	164
	R: AGG CGG TGG ACT CTG GAC TA	
*CMD1*	F: CGC CCA GTG AAG CAG AAG T	190
	R: AAC TCA GCG GCG GAG ATT A	
*CNB1*	F: CTT GCT GGA CGT ATA ATG GAG GT	129
	R: GAA GGC GAA TCT TAA CTT TTC GT	
*PLC1*	F: GAA TGA TAC ATC GCC AAG CAG	181
	R: AGA AAC TTC AGC ATC CCA TAT TG	

a *LexA* primer pairs were used for semi-quantitative RT-PCR analysis. *ACT1* primer pairs were used for both semi-quantitative and quantitative RT-PCR. *PMC1*, *CMD1*, *CNB1*, and *PLC1* primer pairs were used for quantitative RT-PCR.

For quantitative RT-PCR, 0.4 µL of a 5-fold dilution of cDNA from each sample was used to analyze gene expression by the SYBR Premix *Ex Taq* (Takara). The cycling program was as follows: an initial cycle of 5 min at 95°C, followed by 45 cycles of 15 s at 95°C, 10 s at 60°C and 15 s at 72°C. Data were collected and analyzed by the real-time PCR system (Eppendorf realplex2). *ACT1* was used as an internal control. Primers for *PMC1*, *CMD1*, *CNB1* and *PLC1* are shown in Table 1.

### Terminal deoxynucleotidyl transferase-mediated dUTP nick end labeling (TUNEL) analysis

To visualize DNA strand breaks, cells were fixed in 4% formaldehyde in PBS (pH 7.4) then treated with lyticase (Sigma, St. Louis, MO, USA) and stained with fluorescein isothiocyanate-labeled TUNEL reagent (*in situ* cell death detection kit; Roche, Mannheim, Germany) [13], [43]. TUNEL fluorescence was examined at a 488-nm excitation and filtered at 525 nm. Photos were taken under a fluorescence microscope (VANOX-AH-1; Olympus).

### Intracellular Ca2+ measurement

Yeast cells were grown in 1/2 SD/Gal-Raf/-Ura or 1/2 SD/Gal-Raf/-His medium with or without treatment for 6 h. After harvesting, cells were resuspended in PBS (pH 7.4) and vortexed briefly. For determination of intracellular Ca^2+^ levels, yeast cells were incubated in PBS (pH 7.4) at 37°C for 30 min with 10 µM Fluo-3-acetoxymethyl ester (Fluo-3/AM; Biotium, Hayward, CA, USA) prepared with a 1 mM stock solution in dimethyl sulfoxide. A non-cytotoxic detergent, pluronic F-127 (0.1%), was added to increase solubility of the Fluo-3/AM. Fluo-3 fluorescence was measured by FACSCalibur with 488-nm (blue) argon (Becton-Dickinson, San Jose, CA, USA) in the FL1 channel. Data acquisition was performed using CellQuest (3.1f) software and data analysis by ModFit LT (3.0) software (Variety Software House). 2,000 to 10,000 cells were measured for each analysis [13], [44].

### Statistical Analysis

Data were calculated as the mean of results from at least three independent experiments or one representative result of parallel experiments. The Origin 8 program was used for calculation. Error bars represent standard deviation (SD).

## Results

### pmc1 mutant displayed increased sensitivity to Al-induced growth inhibition and Al-induced PCD

**Figure 1:**
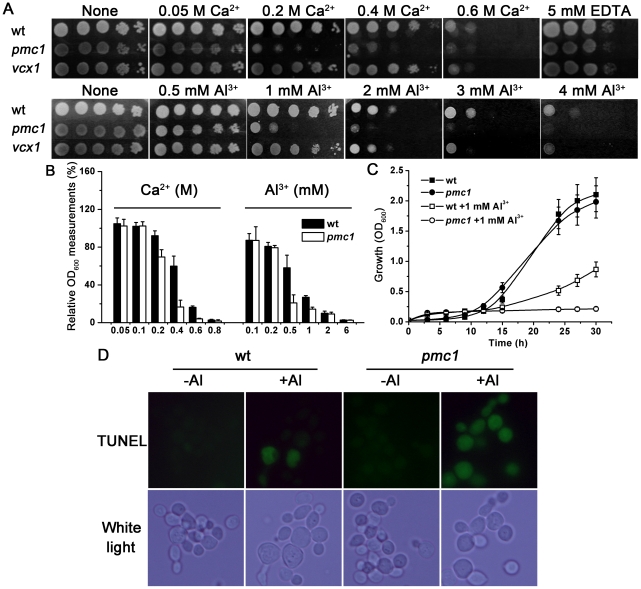
Al sensitivity in the *pmc1* mutant. (A) Growth properties of the wild-type (wt) strain and *pmc1* and *vcx1* mutants under Ca and Al stresses. (B) Comparison of growth (OD_600_) between wt and *pmc1* mutant yeast strains incubated in liquid 1/2 SD/Gal-Raf/-Ura medium containing a series of doses of CaCl_2_ or AlCl_3_ for 24 h. Relative OD_600_ measurements were calculated as the OD_600_ of treated cells divided by the OD_600_ of untreated cells. The value of untreated strains was set at 100%. (C) Yeast wt and *pmc1* strains were incubated in liquid 1/2 SD/Gal-Raf/-Ura medium with or without 1 mM AlCl_3_. The cell densities (OD_600_ values) were determined at 3-h intervals over a 30-h period. (D) Comparison of Al-induced PCD between wt and *pmc1* mutant by TUNEL. No TUNEL signal was detected in the absence of Al treatment

The previous investigations showed that the loss of *PMC1* in yeast cells leads to a failure in pumping Ca^2+^ into the vacuole; thus, the cells failed to survive high Ca^2+^ stress [39]. To examine the roles of vacuolar Ca^2+^ transporters in Al toxicity, we tested the sensitivity of *pmc1* and *vcx1* mutants under a series of exogenous Ca^2+^ and aluminum ion (Al^3+^) treatments. As shown in Figure 1A, upon exposure to Ca^2+^ and Al^3+^, the *pmc1* mutant was more sensitive than wild-type yeast and the *vcx1* mutant. Because of the variant status of other mutants tested under normal conditions as well as their reduced sensitivity to Al treatment (Figure S1), we chose PMC1 as the best candidate for studying Al tolerance.

As shown in Figure 1B, the *pmc1* mutant grew as robustly as the wild-type strain in medium containing low concentrations of Ca^2+^ (0–100 mM) but grew at a much slower pace under conditions of high concentrations of Ca^2+^ (200–800 mM). Furthermore, compared to the wild-type control, the *pmc1* mutant was more sensitive to moderate levels (0.5–1 mM) of Al^3+^ in liquid medium. We then performed a time-course assay to monitor yeast growth with 1 mM Al treatment. As shown in Figure 1C, when comparing the two growth curves generated from conditions of no Al exposure, the mutant cells exhibited a very similar rate to that of wild-type cells. However, the *pmc1* mutant displayed more sensitivity to Al treatment. The difference in growth (OD_600_) appeared early, between the 10^th^–15^th^ h, and became increasingly significant thereafter when mutant cells completely ceased to grow. In addition, both strains exhibited positive effects of Al toxicity within the first 10 h. This result indicated that the loss of vacuolar *PMC1* in the mutant cells led to a failure in the regulative response to Al-elicited Ca^2+^ afflux.

**Figure 2:**
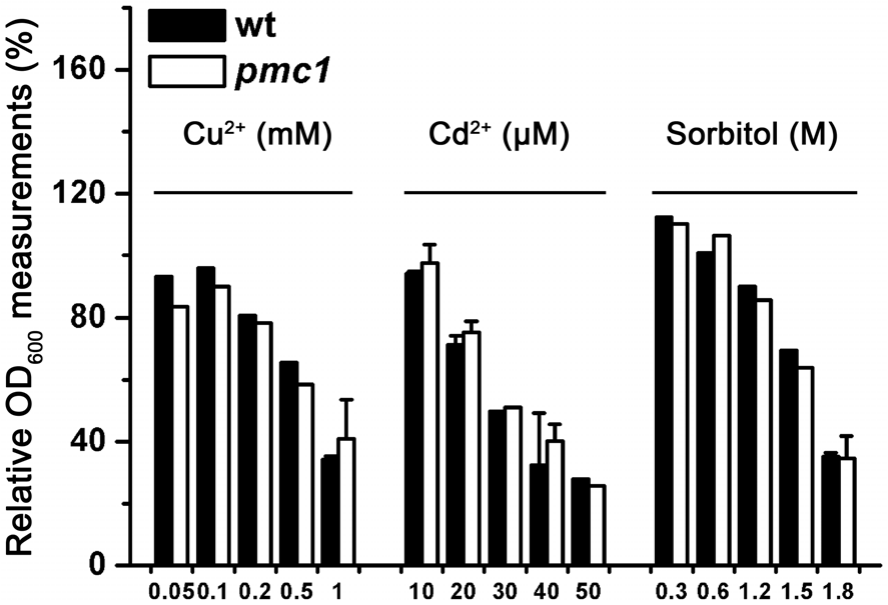
Stress specificity test on the *pmc1* mutant. Comparison of growth (OD_600_) between wt and *pmc1* mutant yeast strains incubated in liquid 1/2 SD/Gal-Raf/-Ura medium containing a series of doses of sorbitol, CuCl_2_ or CdCl_2_ for 24 h. Relative OD_600_ measurements were calculated as the OD_600_ of treated cells divided by the OD_600_ of untreated cells. The value of untreated strains was set at 100%.

To ensure that the Al susceptibility of the *pmc1* mutant was not an artifact, we tested the vulnerable specificity of the *pmc1* mutant. As shown in Figure 2, the yeast cells were exposed to diverse stresses that could induce apoptosis in yeast, including different concentrations of sorbitol or other metal ions, such as copper ion (Cu^2+^) or cadmium ion (Cd^2+^). The *pmc1* mutant displayed enhanced sensitivity to only Ca^2+^ and Al^3+^ but not to any of the other substances.

Taking into account all of these results along with those from our previous studies [13], Al toxicity in yeast appears to be mediated by interrupting the normal cytoplasmic Ca^2+^ pool and, consequently, by activating Ca signaling.

Al can induce both apoptotic-like cell death and an increase of Ca^2+^ level in yeast [13]. However, it remains to be determined whether Ca signals mediate Al-induced PCD. For this purpose, the TUNEL assay was used to detect apoptosis in wild-type yeast and the *pmc1* mutant under Al stress. As shown in Figure 1D, TUNEL-positive cells were observed in both Al-treated strains. Moreover, the number of TUNEL-positive *pmc1* mutant cells was much higher than that of the wild-type cells, indicating Al-elicited Ca signaling is an early mechanism of Al-induced PCD.

### Overexpression of PMC1 reduced Al sensitivity in yeast

**Figure 3:**
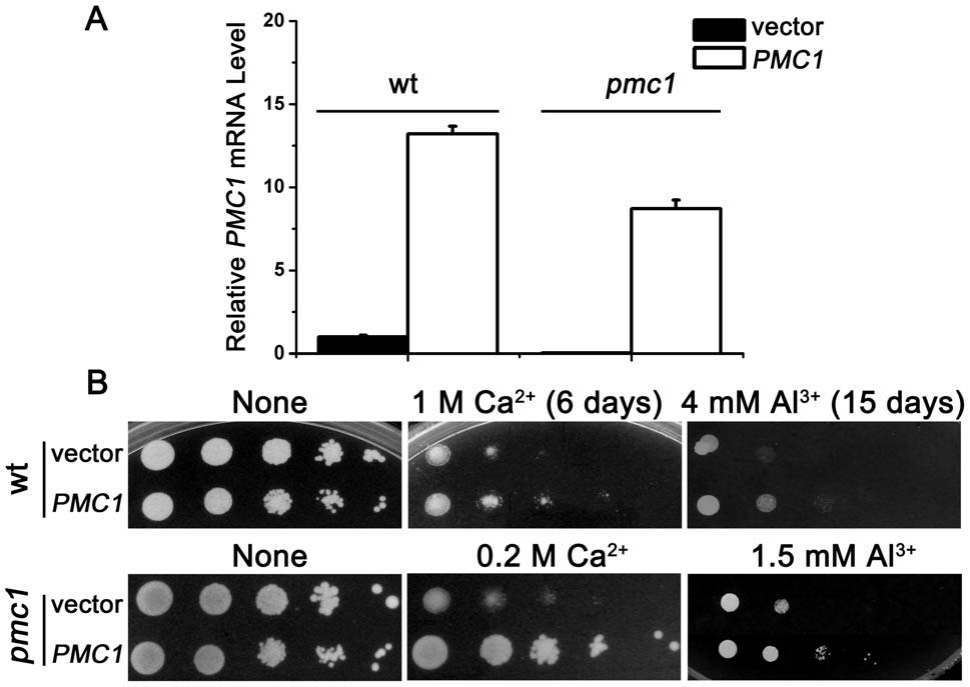
Overexpressed Ca^2+^-ATPase *PMC1* alleviates Al^3+^-induced cell death in yeast. (A) Quantitative RT-PCR analysis of *PMC1* expression in transformants. *ACT1* was the internal standard. (B) Growth properties of transformed wt and *pmc1* mutant strains stressed with Ca^2+^ or Al^3+^. Log-phase cells were diluted in a 10-fold series and spotted on 1/2 SD/Gal-Raf/-Ura plates containing the indicated stresses. The plates were then incubated at 30°C. Photos were taken after three or the indicated number of days of incubation.

To investigate if *PMC1* assumes an Al tolerance function in complementation tests, the exogenous *PMC1* gene was transformed into wild-type yeast or back into the *pmc1* mutant. Quantitative RT-PCR analysis showed that the levels of *PMC1* mRNA in wild-type overexpressing exogenous *PMC1* was 12 times higher than wild-type, and *pmc1* mutant overexpressing *PMC1* exhibited a level of *PMC1* mRNA that was about 8 times higher than wild-type yeast (Figure 3A). Due to the enhanced susceptibility of the *pmc1* mutant, lower concentrations of Ca^2+^ or Al^3+^ were used, compared with those concentrations used for the wild-type strain. As shown in Figure 3B, both wild-type and *pmc1* strains exhibited increased Ca and Al tolerance in response to *PMC1* overexpression.

To examine the causality between Al stress and Ca signals, we tested the alteration of cytosolic Ca^2+^ levels under Al treatment. Flow cytometry studies were performed to quantify Ca^2+^ levels using the Ca-specific probe Fluo-3/AM. As shown in Table 2, cytosolic Ca^2+^ levels were increased in wild-type cells when Al was present in the medium, and this result is consistent with previous studies [13]. Furthermore, the Ca^2+^ levels in wild-type cells overexpressing *PMC1* were distinctly less than wild-type cells transformed with vector only in response to Al^3+^ at all concentrations tested. These data suggest that sequestration of Ca^2+^ into the vacuole for cytoplasmic Ca^2+^ homeostasis was achieved by genetic engineering with the overexpression Ca^2+^-ATPase *PMC1*, which represents a novel approach to the improvement of Al tolerance.

**Table 2:** Alteration of intracellular Ca^2+^ levels by *PMC1*.

Ca^2+^ Responses in Yeast After Stimulation With Different Concentrations of Al^3+^ a			
Ca^2+^ response (fluorescence ratio)^b^			
**Al^3+^(mM)**	**0.5**	**1**	**4**
wt	1.60±0.62	1.78±0.04*	2.52±0.53**
wt/*PMC1*	1.28±0.46	1.40±0.50*	1.91±0.86*

a Data are presented as mean±SD for at least three experiments. * p<0.05, and ** p<0.01 versus the untreated control values

b Fluorescence ratio  =  value of mean FL1 in Al^3+^-stimulated yeast/mean FL1 in Al^3+^-unstimulated yeast. The value of untreated strains was set at 1.

### Al toxicity is reduced by treatment with Ca2+ chelator BAPTA-AM

BAPTA-AM is a membrane permeable compound that is capable of mediating the redistribution of Ca^2+^ throughout diverse intracellular compartments; thus, BAPTA-AM can control cytoplasmic free Ca^2+^ at low levels [45], [46], [47]. To provide direct evidence that Ca^2+^ is an important mediator of Al toxicity, we tested yeast cells with the Ca^2+^ chelator BAPTA-AM to inhibit excessive cytosol free Ca^2+^ and monitored the effects.

**Figure 4:**
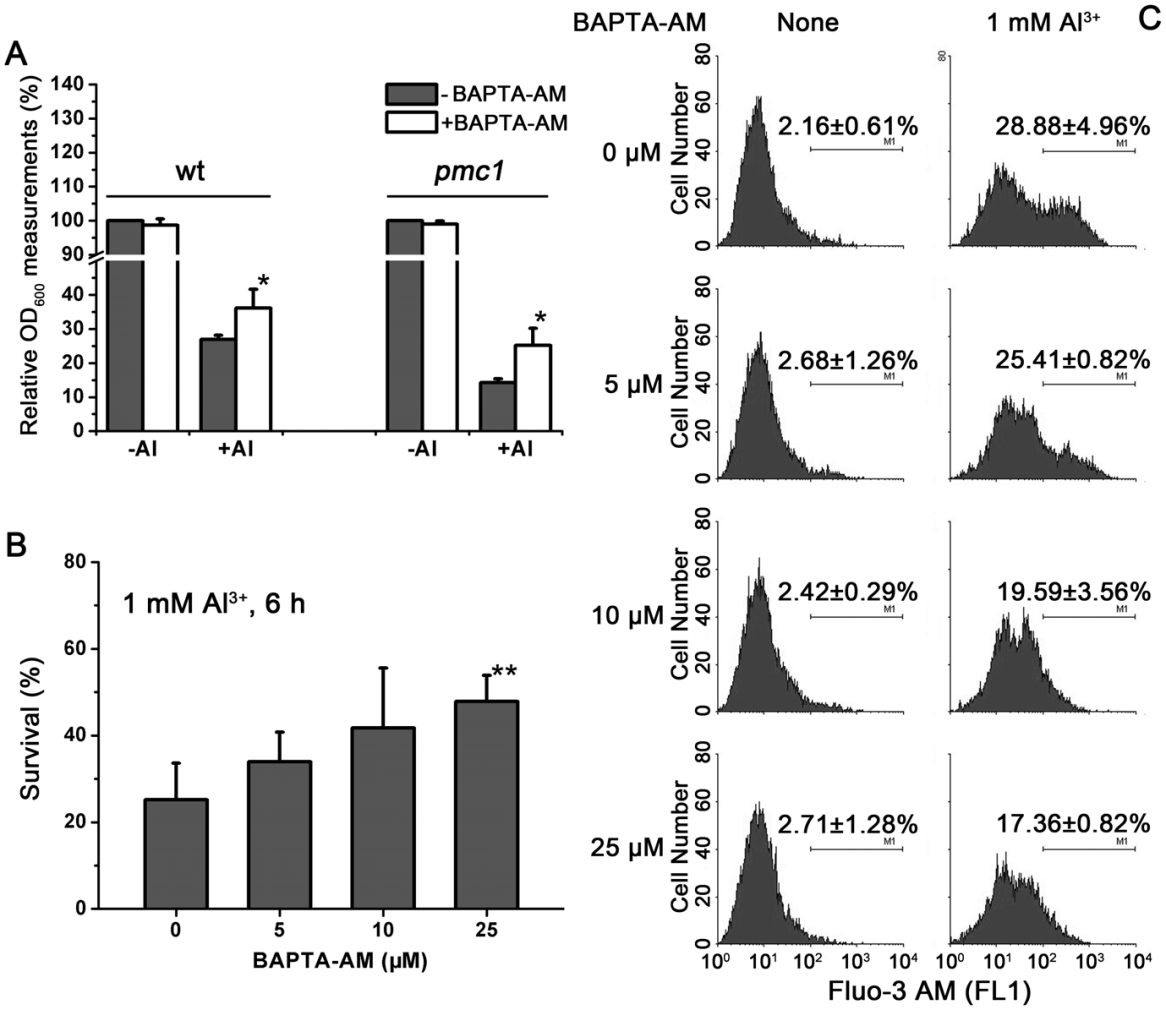
Reduced Al toxicity with the Ca^2+^ chelator BAPTA-AM. (A) Comparison of growth (OD_600_) with or without BAPTA-AM in wt and *pmc1* mutants. Yeast strains were incubated in liquid 1/2 SD/Gal-Raf/-Ura medium containing 1 mM AlCl_3_ with or without BAPTA-AM for 24 h. Relative OD_600_ measurements were calculated as the OD_600_ of treated cells divided by the OD_600_ of untreated cells. The value of untreated strains was set at 100%. (B) Survival tests of yeast cells under Al stress with different concentrations of BAPTA-AM. The yeast cells were pretreated with the indicated concentrations of BAPTA-AM in liquid medium for 30 min, followed by treatment with 1 mM Al^3+^ for 6 h, and then, the cells were plated for survivors on YPD plates. The values were calculated as the percentage of the number of surviving cells without Al treatment. Viability without Al was set at 100%. The results are the means of at least three independent experiments. *p<0.05, and **p<0.01 versus the untreated control values. (C) Al stress-increased cytoplasmic Ca signals can be alleviated by BAPTA-AM. Flow cytometry analysis of 1 mM Al^3+^-challenged cytosolic Ca^2+^ levels in wt yeast.

As shown in Figure 4A, yeast cells grew in the presence of different amounts of chelator BAPTA-AM. Treatment with 25 µM BAPTA-AM appeared to favor cell growth when Al stress was also present. During a 24-h incubation period, both wild-type and *pmc1* mutant cells with BAPTA-AM treatment had higher densities than the cells without BAPTA-AM treatment in the presence of Al^3+^. BAPTA-AM treatment alleviated exogenous Ca^2+^ stress in wild-type cells after about 20 h, whereas in *pmc1* this alleviation occurred at an earlier time point (data not shown). The chelator had little effect on unstressed cells. Furthermore, all the concentrations of BAPTA-AM used in this study effectively enhanced the viability of Al-exposed cells (Figure 4B). These data suggest that treatment with the cytosolic Ca^2+^ chelator BAPTA-AM can alleviate Al toxicity in yeast, directly supporting the idea that Al-induced cell death is mediated by cytosolic Ca signaling flux and Ca^2+^ homeostasis, which are both essential for cell viability.

To further explore the chelator BAPTA-AM function on Ca^2+^ levels in Al tolerance, the changes in cytosolic Ca^2+^ levels were monitored by flow cytometry. As shown in Figure 4C, BAPTA-AM caused a redistribution of cytosolic Ca^2+^ in the cells exposed to Al. The basal cytosolic Ca^2+^ concentrations in unstressed cells remained largely unaffected. When incubated with 1 mM Al^3+^, however, BAPTA-AM exposure led to a dose-dependent decrease in cytosolic Ca^2+^ in cells. Similar to the above results, BAPTA-AM exposure also resulted in a dose-dependent decrease in cytosolic Ca^2+^ under 0.5 mM Al^3+^ or 0.2 M Ca^2+^ treatment (Figure S2). These data are consistent with what we observed in the survival tests and strongly suggest that inhibiting the Al-elicited Ca^2+^ burst can reduce Al toxicity.

### Heterogeneous anti-apoptotic proteins improved Al tolerance of the pmc1 mutant

**Figure 5:**
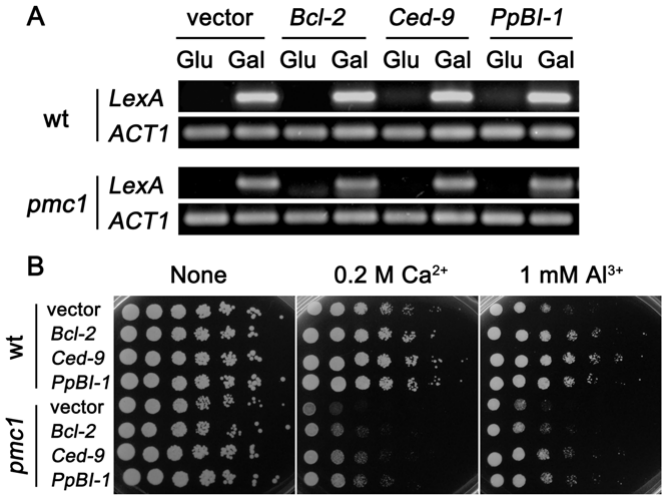
Heterogeneous anti-apoptotic members improve the Al tolerance of the *pmc1* mutant. (A) Semi-quantitative RT-PCR analysis of the expression of anti-apoptotic members in the wt and *pmc1* mutant strains after incubation in induced (Gal) or uninduced (Glu) medium. *ACT1* served as the internal standard. (B) Growth properties of yeast cells transformed with anti-apoptosis members under Ca and Al stresses. Log-phase cells were diluted in a 5-fold series with an initial OD_600_ of 1, and then, 5 µL of each dilution was spotted on 1/2 SD/Gal-Raf/-His plates containing different stresses. Photos were taken after three days of incubation at 30°C.

Previous studies showed that the Al-triggered Ca^2+^ level increase could be blocked by anti-apoptotic proteins [13]. This result led us to test whether the expression of anti-apoptotic members restore Al tolerance in the *pmc1* mutant. As described before, all three transformants were morphologically indistinguishable, and their growth rates were similar. Semi-quantitative RT-PCR showed equally abundant expression of the three transgenes in induced medium (Gal) but almost no PCR signal in uninduced medium (Glu) (Figure 5A). As shown in Figure 5B, the strains expressing *Bcl-2*, *Ced-9* or *PpBI-1* exhibited robust growth under both Ca and Al stresses, suggesting that the negative regulation of PCD alleviated Al toxicity through the regulation of Ca homeostasis.

We examined the alteration of cytosolic Ca^2+^ levels in the wild-type, *pmc1* mutant and transgenic cells expressing anti-apoptotic members under Al stress. Al^3+^ treatment (1 mM) resulted in increased Ca^2+^ levels in both wild-type and *pmc1* mutant cells compared to the unstressed cells. Al-induced cytoplasmic Ca^2+^ levels in cells expressing anti-apoptotic members was distinctly decreased compared to the cells transformed with vector only (Table 3), suggesting that anti-apoptotic members can complement the function of Ca^2+^-ATPase *PMC1* and act upstream of intracellular Ca^2+^ flux in the pathway mediating Al-induced cell death.

**Table 3:** Alteration of intracellular Ca^2+^ levels by anti-apoptotic members.

Ca^2+^ Responses in Yeast After Stimulation With 1 mM Al^3+^ a				
Ca^2+^ response(fluorescence ratio) b				
	**vector**	**Bcl-2**	**Ced-9**	**PpBI-1**
**wt**	1.72±0.23**	1.58±0.27	1.47±0.08*	1.35±0.39
***pmc1***	1.32±0.12*	0.95±0.21	1.16±0.27	0.91±0.18

a Data are presented as mean±SD for at least three experiments. * p<0.05, and ** p<0.01 versus the untreated control values

b Fluorescence ratio  =  value of mean FL1 in Al^3+^-stimulated yeast/mean FL1 in Al^3+^-unstimulated yeast. The value of untreated strains was set at 1.

### Ca pathway components participate in Al tolerance

As shown above, regulating cytosolic Ca^2+^ significantly improved Al tolerance in yeast cells. To investigate the molecular mechanisms underlying Ca signaling, we determined the expression levels of certain Ca pathway components using quantitative RT-PCR. Three strains were selected for this assay: wild-type cells, cells pretreated with BAPTA-AM, and cells transformed with *Ced-9*.

**Figure 6:**
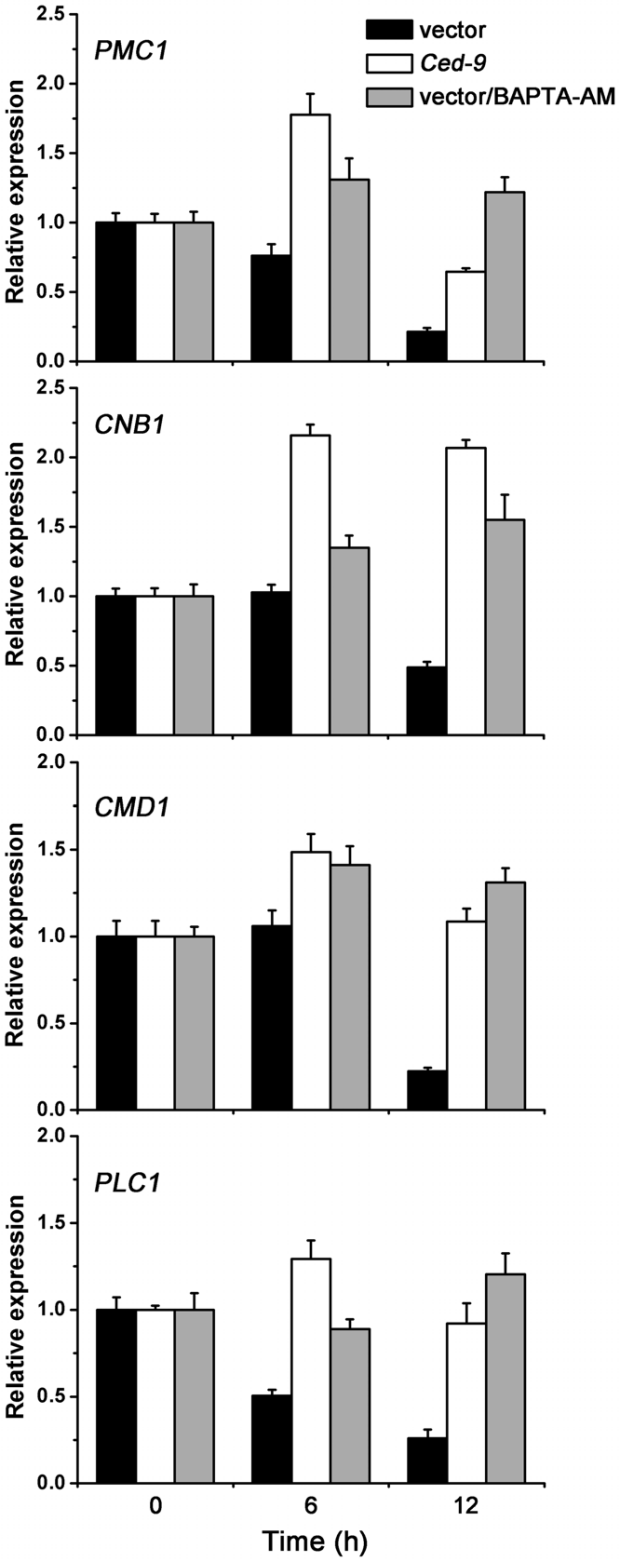
Ca pathway components participate in Al tolerance of yeast. wt/pGilda, wt/pGilda-*Ced-9* and wt/pGilda/BAPTA-AM were treated with or without Al^3+^. The relative expression levels of genes were determined by quantitative RT-PCR. *ACT1* was used as an internal control. 0 h represents yeast with Al treatment at 0 h. Data are the mean ± SD (n = 6; two biological replicates and three PCR replicates).

Figure 6 shows that accumulation of *PMC1* mRNA is significantly reduced when cells are exposed to Al. Expression levels of *PMC1* in *Ced-9* transformants or BAPTA-AM pretreated cells increased by about 3- or 6-fold over the wild-type cells when exposed to Al^3+^ for 12 h. Consistent with Al sensitivity of the *pmc1* mutant, these results suggest that cytosolic Ca^2+^ homeostasis and *PMC1* activation are important for Al tolerance.

Small variations in cytosolic Ca^2+^ that occur in response to a number of stimuli are sufficient to activate a variety of Ca-sensing proteins, such as CaM and calcineurin; this activation then leads to the induction of various downstream signal transduction pathways [24], [36]. The expression of *CMD1* (which encodes CaM) and *CNB1* (which encodes the regulatory subunit of calcineurin) decreased upon exposure to Al (Figure 6). It has been reported that PLC activity is inhibited by Al in plants [37]. Our data showed a similar result for *PLC1*, which encodes PLC and was down-regulated when cells were exposed to Al^3+^. However, the expression of *CMD1*, *CNB1* and *PLC1* were restored to normal or even higher levels in *Ced-9* transgenic colonies and BAPTA-AM pretreated cells. These results indicate that these Ca^2+^-relevant genes in Ca signaling pathways participate in Al tolerance in yeast.

## Discussion

In this study, we investigated the relevance of a vacuole-located Ca^2+^-ATPase PMC1 in modulating Al stress responses and the function of cytoplasmic Ca homeostasis on Al tolerance in yeast. When compared to wild-type, the *pmc1* mutant exhibited more sensitivity to Al stress (Figure 1), as well as to high Ca^2+^ concentrations [39]. Al induces apoptosis in yeast via the elevation of cytosolic Ca^2+^, which is released from intracellular sources, and apoptotic suppressors enhance Al tolerance with decreased Ca signals [13]. PMR1, a Golgi Ca^2+^/Mn^2+^-ATPase, plays a role in Al tolerance [48]. In particular, PMR1 is important for intracellular Ca homeostasis, and PMC1 transcription increases in the *pmr1* mutant to sequester cellular Ca^2+^ into intracellular stores [49], suggesting that Ca^2+^-mediated signaling plays a pivotal role in Al tolerance of yeast.

Since the first description of apoptosis in yeast [17], many factors, including sorbitol [50], Al [13], Cu [51], Cd [52] and others, have been found to induce yeast apoptosis. Sorbitol and Cu both trigger apoptosis via a mitochondrial pathway, with caspase and cytochrome *c* occasionally involved [50], [51]. Cd^2+^ induces ER and oxidative stresses in yeast. Cd toxicity is a direct consequence of Cd^2+^ accumulation in the ER [52], [53], [54]. In particular, PMR1 has a central role in the regulation of intracellular levels of Cd^2+^ and Cd^2+^ detoxification [55]. By contrast, *pmc1* mutant and wild-type cells show the same tolerance to CdCl_2_ [55], which is consistent with our results. These results clearly indicate that PMC1 plays selective roles in Al tolerance as opposed to the other stresses we tested.

The addition of Al to cultures changes pH values. However, based on our experimental data, Ca^2+^ changed little in response to pH variations, which was different with Al^3+^ (Figure S3A). Particularly when the pH was 4.0 or 3.8, both 0.5 mM Al and 1 mM Al medium, which we often use, have a similar pH. Ca^2+^ level in the *pmc1* mutant was elevated when the pH reached 3.4. The proton (H^+^) concentration may be too high and may affect both the H^+^ gradient and the pH of the vacuole or other organelles. The growth properties of wild-type yeast and the *pmc1* mutant were similar when cells were exposed to different pH values (Figure S3B), whereas the *pmc1* mutant was more sensitive to Al than wild-type yeast. These results suggest that the differences between the *pmc1* mutant and wild-type yeast under Al stress are caused by Al^3+^ rather than pH change.

Al^3+^ exposure can elicit a striking and rapid increase in cytosolic Ca^2+^ in plants and yeast [2], [13]. Ca^2+^ transport across the plasma membrane and intracellular sequestration are tightly regulated events that maintain the cytosolic Ca^2+^ concentrations within a range of 50–200 nM [49]. As such, the regulation of intracellular Ca homeostasis in eukaryotic cells is a remarkably intricate and dynamic process, which led us to explore the relation between cytosolic Ca^2+^ fluctuation and Al tolerance. PMC1 overexpression enhanced Al and Ca tolerance in wild-type yeast and the *pmc1* mutant (Figure 3). Endomembrane Ca^2+^-ATPases appear to be important for intracellular Ca^2+^ distribution [28], [40], [56]. Recent studies have shown that PMCA overexpression depletes intracellular Ca^2+^ stores and induces apoptosis through the mitochondrial pathway in clonal β-cells [57]. Because PMCA and PMC1 are localized to different regions, we hypothesized that the vacuole was the key Ca^2+^ store involved in regulating cytosolic Ca^2+^ in response to Al stress in yeast. In addition to Ca^2+^ and Al^3+^, we also found that the *pmc1* mutant displayed sensitivity to H_2_O_2_ (Figure S4), which suggested that Ca signals are related reactive oxygen species (ROS) production [58]. A recent study also showed that lethal H_2_O_2_ shock predominantly mobilized the vacuolar Ca^2+^ in yeast [59]. The studies of Ca^2+^-ATPases *AtACA4* and *AtACA11* in plant also indicate that endomembrane Ca^2+^ pumps function as suppressors of a salicylic acid-dependent PCD pathway and that vacuoles can modulate Ca signals [60].

When subjected to Al stress, the cytosolic Ca^2+^ concentration in PMC1 transformants was lower than in wild-type cells (Table 2). Down-regulation of Al-elicited cytosolic Ca^2+^ enhanced Al tolerance. Buffering cytosolic Ca^2+^ with BAPTA-AM resulted in the inhibition of apoptosis, which would have otherwise been caused by oxidative stress [45], Cd [46], [47], Galectin-9 [61], and various other conditions [62], [63], [64]. Consistent with the BAPTA-AM-induced down-regulation of cytosolic Ca^2+^, the elevation of cell death stimulated by Al was abolished (Figure 4). These results indicated that Ca oscillation played a pivotal role in mediating Al toxicity. In particular, the decrease of cytoplasmic free Ca^2+^ may be one mechanism that reduces Al toxicity.

Bcl-2 family and BI-1 proteins regulate intracellular Ca^2+^ homeostasis [65], [66], [67]. Earlier studies have shown that Bcl-2 overexpression leads to increased Sarco/endoplasmic reticulum Ca^2+^-ATPase (SERCA) expression [68], [69]. Bcl-2, Ced-9 and PpBI-1 significantly improve Al tolerance and block Al-elicited Ca signals [13]. Ced-9 inhibits Al-induced activity of caspase-like vacuolar processing enzyme (VPE) [12]. These apoptotic suppressors restored both wild-type and *pmc1* mutant growth under Al and Ca stresses (Figure 5). We chose 1/2 SD instead of full SD because yeast growth exhibited more Al sensitivity and the Bcl-2 gene enhanced Al tolerance in this medium. Cytosolic Ca signal levels detected in wild-type and *pmc1* mutant cells expressing apoptotic suppressors were distinctly less than the levels in the cells transformed with vector only after exposure to 1 mM Al^3+^ (Table 3), suggesting that anti-apoptotic members complemented the function of Ca^2+^-ATPase PMC1 and enhanced Al tolerance to some extent.

Our study demonstrates, for the first time, that Al toxicity is mediated by Ca signals and that the vacuolar Ca^2+^-ATPase Pmc1p plays a pivotal role in Al tolerance in yeast. These findings may make it possible to genetically improve Al tolerance in plant species by protecting intracellular Ca^2+^ homeostasis.

## Supporting Information

Figure S1 **Effect of Ca and Al stresses on the cell growth of** ***pmr1*** **and** ***pmc1 pmr1 cnb1*** **mutants.** Growth properties of wt and mutant strains under Ca (A) and Al (B) stresses.
(TIF)

Figure S2 **Al and Ca stress-increased cytoplasmic Ca signals can be alleviated by BAPTA-AM.** Flow cytometry analysis of 0.5 mM Al^3+^- and 0.2 M Ca^2+^-challenged cytosolic Ca^2+^ levels in wt yeast.
(TIF)

Figure S3 **Ca signals and growth properties of wt and** ***pmc1*** **mutant strains in response to pH variations.** A. Ca^2+^ changed little in response to pH variations. Flow cytometry analysis of pH-challenged cytosolic Ca^2+^ levels in wt and *pmc1* mutant strains. B. Growth properties of yeast cells under different pH values.
(TIF)

Figure S4 **H_2_O_2_ sensitivity in the** ***pmc1*** **mutant.** Growth properties of the wt strain and the *pmc1* mutant under H_2_O_2_ stress.
(TIF)
