# Supplementary figures and images for: Regulating Cytoplasmic Calcium Homeostasis Can Reduce Aluminum Toxicity in Yeast
Source: PLoS One. 2011 Jun 15;6(6):e21148. doi: 10.1371/journal.pone.0021148 (PMC3115986; doi:10.1371/journal.pone.0021148)

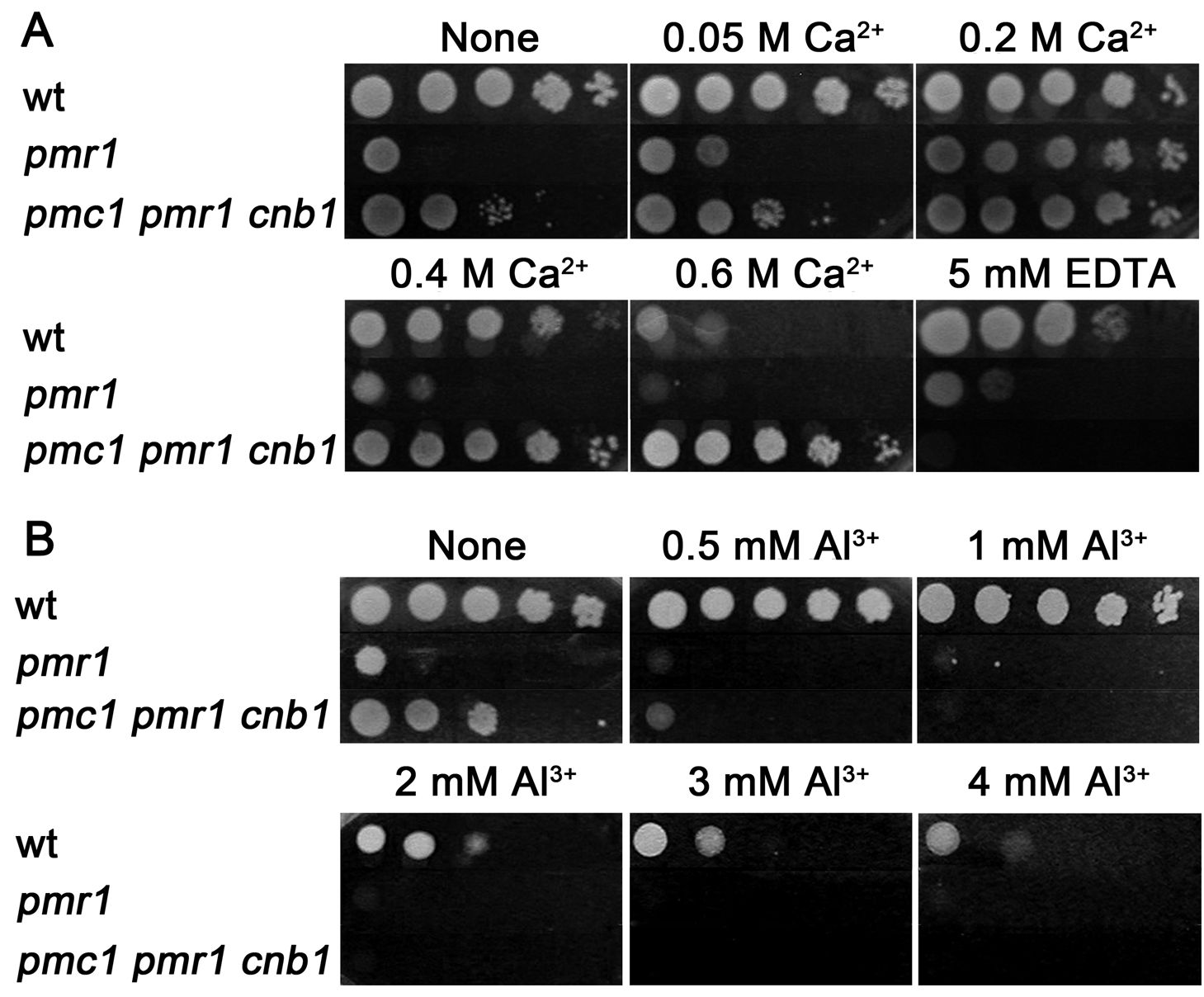

Supplement: Figure S1 — Effect of Ca and Al stresses on the cell growth of pmr1 and pmc1 pmr1 cnb1 mutants. Growth properties of wt and mutant strains under Ca (A) and Al (B) stresses. (TIF) [file pone.0021148.s001.tif]

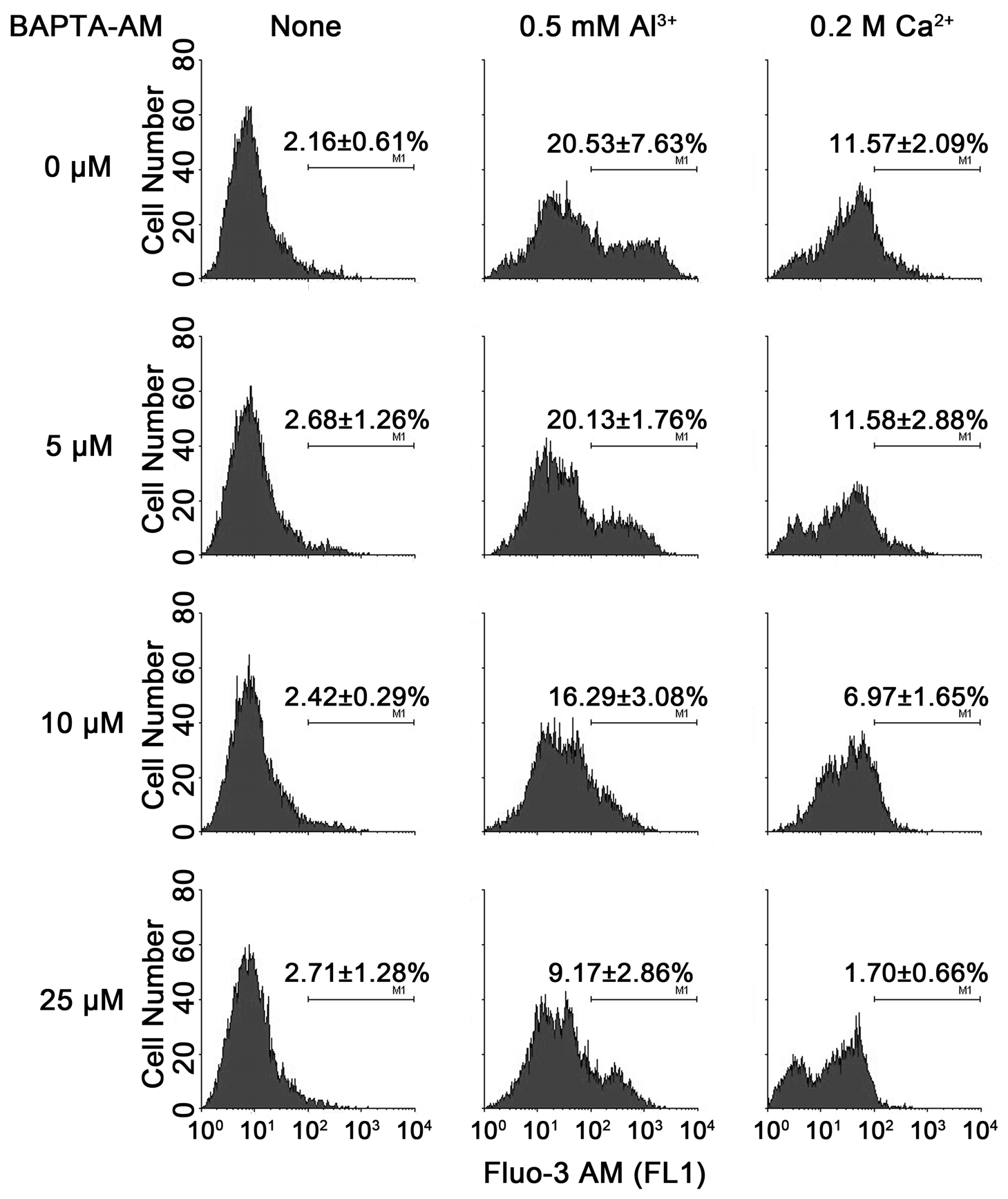

Supplement: Figure S2 — Al and Ca stress-increased cytoplasmic Ca signals can be alleviated by BAPTA-AM. Flow cytometry analysis of 0.5 mM Al3+- and 0.2 M Ca2+-challenged cytosolic Ca2+ levels in wt yeast. (TIF) [file pone.0021148.s002.tif]

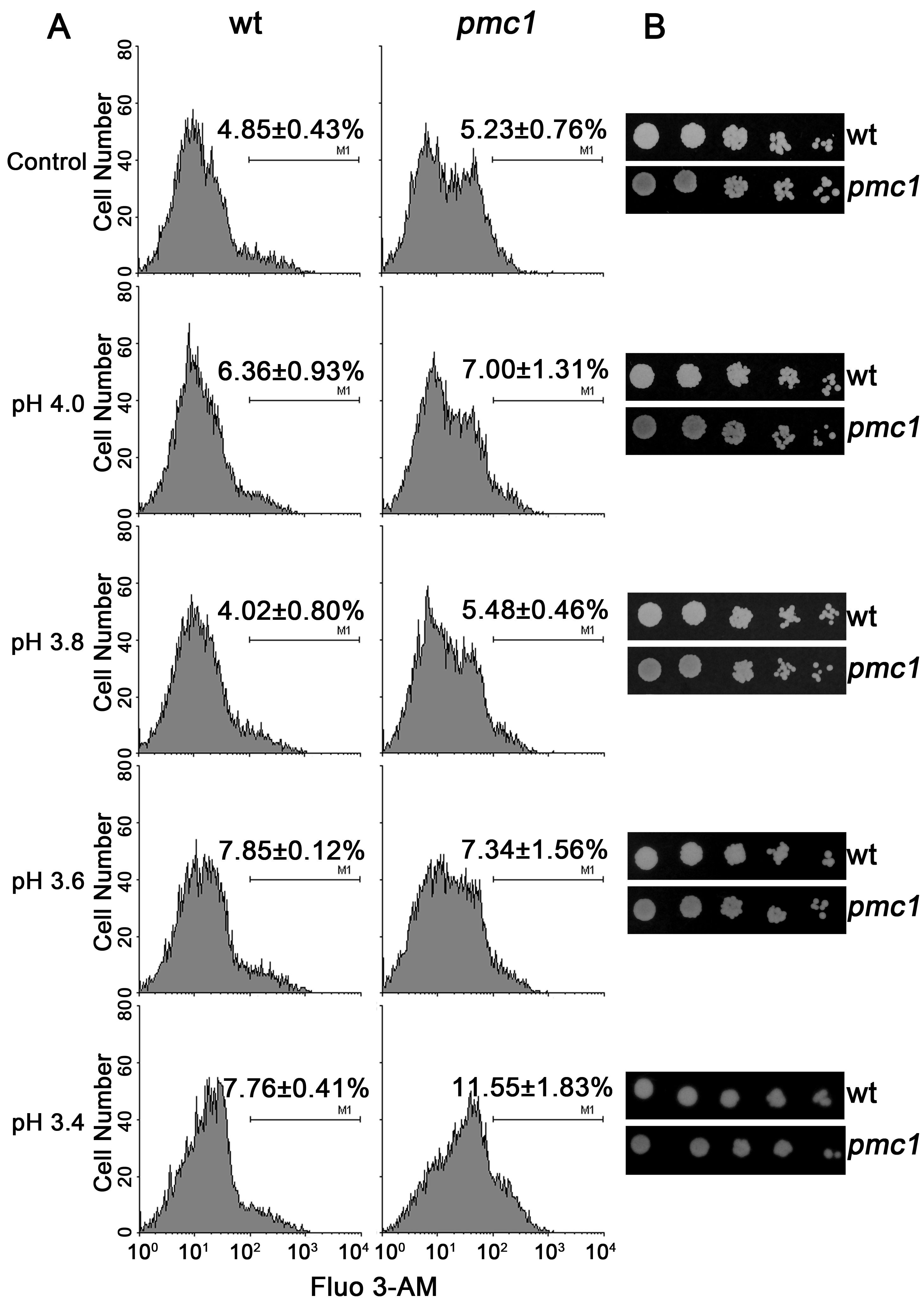

Supplement: Figure S3 — Ca signals and growth properties of wt and pmc1 mutant strains in response to pH variations. A. Ca2+ changed little in response to pH variations. Flow cytometry analysis of pH-challenged cytosolic Ca2+ levels in wt and pmc1 mutant strains. B. Growth properties of yeast cells under different pH values. (TIF) [file pone.0021148.s003.tif]

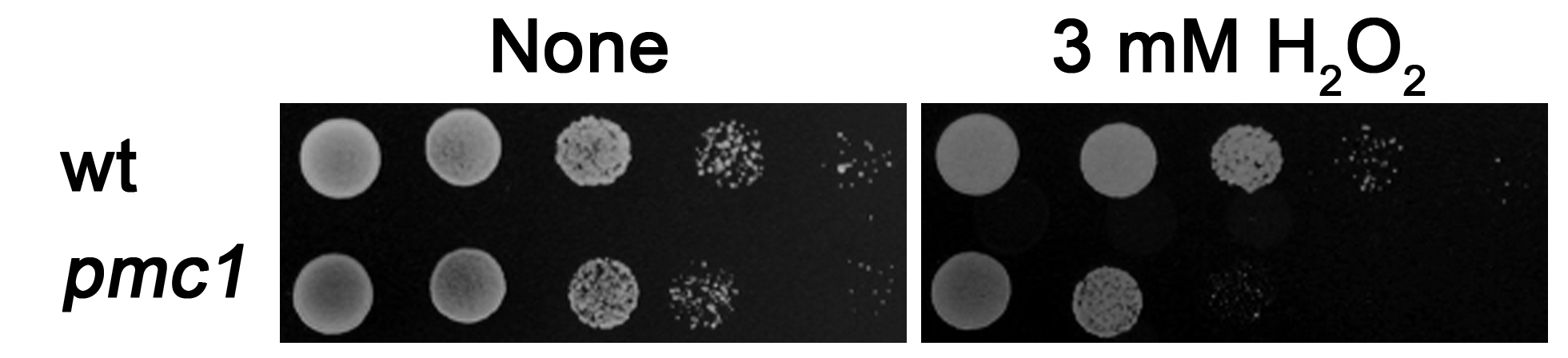

Supplement: Figure S4 — H2O2 sensitivity in the pmc1 mutant. Growth properties of the wt strain and the pmc1 mutant under H2O2 stress. (TIF) [file pone.0021148.s004.tif]
